# The Stapler Dilemma in VATS Wedge Resection: Are Sutures a Viable Alternative?

**DOI:** 10.3390/jcm14207356

**Published:** 2025-10-17

**Authors:** Mithat Fazlioglu, Argun Kıs, Gokhan Ozturk, Nevin Fazlioglu

**Affiliations:** 1 Departmant of Thoracic Surgery, Medical Faculty, Tekirdag Namik Kemal University, 59030 Tekirdağ, Turkey; 2 Departmant of Thoracic Surgery, Medical Faculty, Pamukkale University, 20160 Pamukkale, Turkey; argunkis@yahoo.com (A.K.); drgokhanozturk9@gmail.com (G.O.); 3 Departmant of Pulmonology, Medical Faculty, Tekirdag Namik Kemal University, 59030 Tekirdağ, Turkey; nfazlioglu@nku.edu.tr

**Keywords:** VATS, wedge resection, stapler, clamp-and-suture, cost-effectiveness, operative outcomes

## Abstract

**Background:** This single-center, retrospective, non-randomized observational study aims to explore the outcomes of video-assisted thoracoscopic surgery (VATS) wedge resection using the traditional clamp-and-suture technique versus staplers, with a focus on cost-effectiveness, operative time, and short-term postoperative outcomes. **Methods:** Data from 59 patients who underwent VATS wedge resection between 2018 and 2024 were retrospectively analyzed. Patients were divided into the stapler group (S-group, n = 27) and the clamp-and-suture group (C-group, n = 32). Technique selection was made intraoperatively by the surgeon based on lesion characteristics. Co-primary outcomes were total hospitalization cost and air leak duration > 2 days. Secondary outcomes included drainage time, complications, and hospital stay. The researchers conducted multivariable regression and sensitivity analyses to handle selection bias and confounding variables. Statistical analyses were performed with a significance level of *p* < 0.05. This study was approved by the Tekirdağ University Faculty of Medicine Ethics Committee (Approval No: 2024.22.02.06). **Results:** The C-group lesions showed proximity to the pleural surface at 5 mm compared to 8 mm (*p* = 0.048), indicating significant selection bias. Operation time was longer in the C-group (70 vs. 60 min, *p* = 0.115). Air leak duration and drainage time were similar between groups (*p* = 0.872, *p* = 0.176). Complication rates classified by Clavien–Dindo scale and hospital stay were comparable. The C-group showed reduced hospitalization expenses ($191.6 vs. $371.7) after adjusting for lesion characteristics and confounders while the clinical results between groups remained equivalent (adjusted OR for air leak: 0.68, 95% CI: 0.13–3.51, *p* = 0.645). The cost advantages persisted through sensitivity analysis which tested for selection bias effects. **Conclusions:** The clamp-and-suture method appears to offer a potentially cost-effective alternative to staplers for carefully selected peripheral lesions in VATS wedge resection, particularly in resource-limited settings. The preliminary results need to be treated as speculative because the study uses a non-randomized retrospective design with limited data from a small number of patients treated by one surgeon and shows evidence of selection bias. The obtained results do not qualify as practice-changing recommendations. The validation of these findings requires prospective randomized controlled trials with predetermined selection criteria and extended follow-up periods to establish clinical recommendations.

## 1. Introduction

The increasing detection of solitary pulmonary nodules and lung diseases requiring surgical intervention has led to a rise in video-assisted thoracoscopic surgery (VATS) [1,2,3,4]. While VATS has clear advantages over thoracotomy, including reduced pain and shorter hospital stays, the widespread use of expensive surgical instruments, particularly staplers, has increased healthcare costs [5,6]. Various alternative techniques have been explored to reduce costs, including the use of energy devices, laser ablation, and endoscopic loop ligation. However, one of the oldest and most cost-effective methods—using a non-crushing clamp and primary continuous suturing—remains underutilized in modern thoracic surgery [7,8,9,10].

This study retrospectively examines the feasibility, safety, and cost-effectiveness of clamp-and-suture wedge resection compared to stapler use and aims to explore whether the clamp-and-suture technique may provide comparable short-term outcomes to staplers while offering potential cost savings, particularly in resource-limited settings. 

## 2. Patients and Methods

### 2.1. Study Design and Ethical Approval

**Figure 1:**
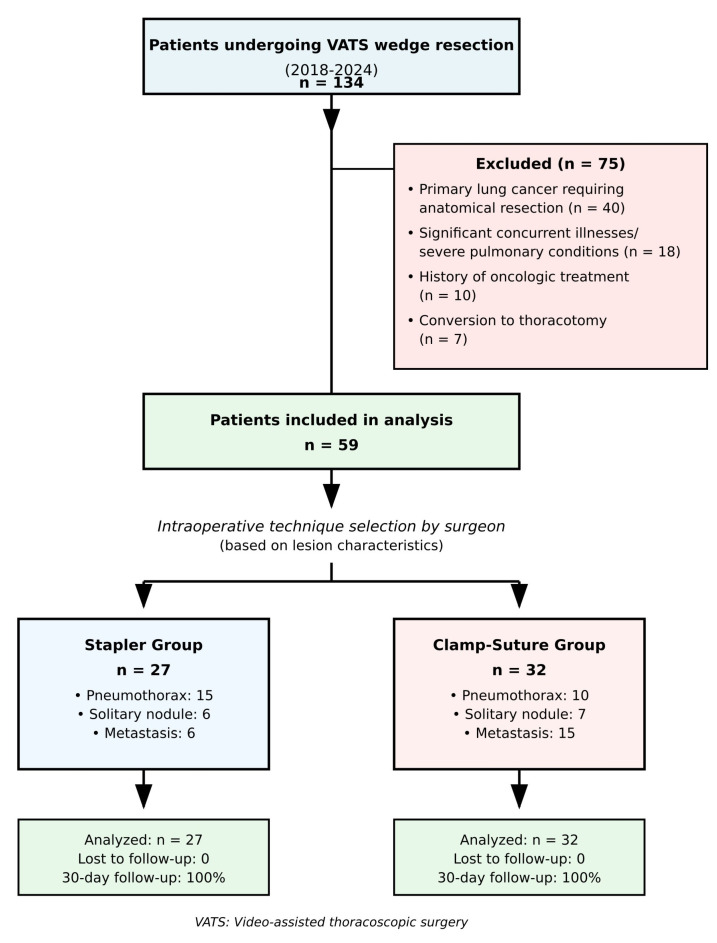
**Flow diagram of patient selection and allocation**. The study examined patient enrollment and exclusion criteria and group assignment for VATS wedge resection procedures between 2018 and 2024. Non-randomized allocation based on intraoperative assessment of lesion characteristics. VATS: Video-assisted thoracoscopic surgery.

This retrospective, single-center, non-randomized observational study was approved by the Ethics Committee of Tekirdağ University of Medicine Faculty (No: 2024.22.02.06, approved on 27 February 2024). The data of patients who underwent video-assisted thoracoscopic surgery (VATS) wedge resection at our institution between 2018 and 2024 were retrospectively analyzed. As a retrospective observational study, no formal sample size calculation was performed. All eligible patients who met the inclusion criteria during the study period (2018–2024) were included to maximize the representativeness and reliability of the data (Figure 1). Written informed consent for surgical procedures was obtained from all patients as part of routine clinical care. The researchers performed a retrospective analysis of anonymized data through procedures that followed institutional guidelines and the principles of the Declaration of Helsinki.

### 2.2. Patient Selection Criteria

Patients who underwent VATS wedge resection for solitary or multiple peripheral pulmonary nodules and pulmonary disease were included in the study. Patients diagnosed with primary lung cancer using frozen sections after wedge resection and subsequent anatomical lung resection were excluded, as the study aimed to examine outcomes of wedge resection performed using either clamps and sutures or staplers.

Other exclusion criteria included

- Patients with significant concurrent illnesses or medical conditions.
- Presence of diffuse pulmonary bullae or severe emphysema identified on chest CT due to potential complications.
- Patients with a history of oncologic treatment due to potential variability in treatment effects on lung tissue.
- Patients requiring conversion to thoracotomy for any reason, as they fell outside the scope of the study.

The study included patients who developed spontaneous pneumothorax through small apical bleb ruptures because these cases can be treated with wedge resection surgery but excluded patients with widespread bullous emphysema because their condition presented higher surgical challenges.

### 2.3. Primary and Secondary Outcomes

The research established two main outcome measures which consisted of (1) complete hospitalization expenses and (2) extended air leak periods beyond two days. The study evaluated additional outcomes through drainage time exceeding three days and postoperative complications and prolonged air leak duration beyond seven days and hospital stays longer than four days and 30-day readmission rates. The exploratory nature of this retrospective study along with unadjusted multiplicity requires all secondary endpoint results to be treated as exploratory findings rather than confirmatory evidence.

### 2.4. Surgical Techniques and Grouping

All patients underwent surgery under general anesthesia with double-lumen tube intubation. The same anesthetic and pain control strategies were used for all patients, without preemptive local or epidural anesthesia. No enhanced recovery after surgery (ERAS) protocol was implemented during the study period. The patients received standardized postoperative pain management through intravenous paracetamol (1 g every 6 h) and nonsteroidal anti-inflammatory drugs according to their needs. Operations were performed by the same thoracic surgeon. A single uniportal VATS approach was used, with a 4–5 cm incision made in the fourth or fifth intercostal space, depending on the location of the lesion.

The uniportal VATS approach was preferred due to its association with reduced postoperative pain, shorter hospital stay, improved cosmetic outcomes, and similar oncologic efficacy compared to multiportal VATS. In wedge resections, especially for peripheral nodules, uniportal access provides sufficient exposure while minimizing intercostal trauma.

Preoperative localization of small or deeply located nodules was performed using transthoracic methylene blue injection under CT guidance in selected cases. The marking procedure was conducted on 8 patients (13.6%) who had nodules measuring less than 10 mm in size or positioned more than 20 mm beneath the pleural surface. The injection was performed within 1 h prior to surgery. No complications related to marking procedures including pneumothorax or hemorrhage or dye diffusion were detected. Intraoperatively, these marked nodules were easily identified through thoracoscopic inspection. In cases without dye marking, nodules were located by direct visualization and gentle palpation of the deflated lung using curved thoracoscopic instruments introduced through the uniport.

Following thoracoscopic exploration of the parenchyma, wedge resection was performed if an abnormal lung area was detected. If a solitary pulmonary nodule or pulmonary metastasis was identified after gentle palpation of the deflated lung, the corresponding area was resected. Patients were divided into two groups based on the technique used for wedge resection:

**Stapler Group (S-group):** Wedge resection was performed using an endoscopic linear cutter stapler, which simultaneously applied multiple rows of staples while cutting the parenchyma.

**Figure 2:**
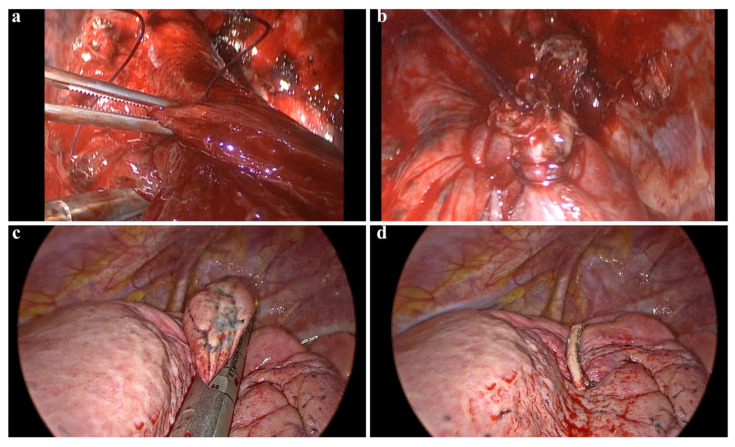
**Surgical technique and postoperative management in VATS wedge resection**. (**a**,**b**) Demonstrate the lung parenchyma being grasped with a clamp and subsequently sutured following a wedge resection. This clamp-assisted suturing technique can be utilized in atypical locations where the placement of an automatic stapler device is challenging. (**c**,**d**) Illustrate images of a wedge resection performed using an endoscopic parenchymal stapler device.

**Clamp-and-Suture Group (C-group):** Wedge resection was performed using a non-crushing clamp. The clamp was closed gradually, and the parenchymal lesion was excised using a scalpel. The resected lung tissue was then sutured using a U-shaped continuous running suture with 3-0 polyglactin sutures. After completing the suture, the clamp was removed, and aerostasis was tested (Figure 2a).

### 2.5. Technique Selection Criteria

The choice between the stapler and the clamp-and-suture technique was determined intraoperatively by the operating surgeon based on the location, size, and accessibility of the lesion, as well as the overall condition of the lung parenchyma. There was no predefined randomization protocol, which represents a significant limitation of this study and introduces potential selection bias. Specific selection criteria included the following:

- Clamp-and-suture technique was generally preferred for
  - Peripheral lesions within 10 mm of the visceral pleura.
  - Lesions ≤ 20 mm in diameter.
  - Areas with minimal pleural adhesions.
  - Cases where cost minimization was a priority.
- Stapler technique was generally selected for
  - Deeper lesions (>10 mm from pleural surface).
  - Larger lesions (>20 mm).
  - Areas with significant adhesions or emphysematous changes.
  - Multiple wedge resections in the same lobe.

This pragmatic selection aimed to ensure patient safety and procedural efficiency while minimizing unnecessary costs. The non-randomized allocation based on surgeon judgment creates indication bias which restricts direct comparison between the groups.

### 2.6. Postoperative Drainage

After resection, either a 24 Fr conventional chest tube or a 12 Fr Hemovac drain was inserted through the same incision used for VATS before closing the wound layers (Figure 2b). The choice of drainage system was based on the extent of resection and anticipated air leak: 24 Fr chest tubes were used for larger resections or when significant air leak was anticipated, while 12 Fr Hemovac drains were used for smaller, peripheral resections with minimal air leak risk. The drainage system was connected to a water-sealed chamber. In cases where lung expansion was incomplete or there was persistent air leakage, negative suction (−20 cmH_2_O) was applied to the drainage system.

### 2.7. Postoperative Management and Follow-Up

Postoperative chest radiography was performed within three hours of transfer to the ward to assess lung expansion (Figure 2c). On postoperative day one, chest tubes were removed in patients who met the following criteria:

- No pleural effusion or pneumothorax on chest radiograph.
- No air leakage detected.
- Daily drainage volume of <150 mL.

Patients were discharged on the same day after chest tube removal, with a follow-up chest radiograph scheduled one week later to assess lung expansion and pleural effusion. All patients were followed for a minimum of 30 days postoperatively to capture any delayed complications or readmissions.

### 2.8. Definition of Prolonged Air Leak and Reinsertion Criteria

Prolonged air leak was defined as persistent air leakage beyond postoperative day seven [11]. In patients with persistent air leaks, bedside air leak testing was performed by submerging the end of the chest tube in water while asking the patient to cough. If visible bubbling was observed, an air leak was confirmed. Patients with prolonged air leaks were discharged with a Heimlich valve for outpatient management. If persistent lung collapse was observed despite suction, reinsertion of a chest tube was performed.

### 2.9. Statistical Analysis

Data analysis was performed using the Statistical Package for the Social Sciences (SPSS) software (version 23.0; IBM Corp., Armonk, NY, USA) and R software (version 4.3.0; R Foundation for Statistical Computing, Vienna, Austria) for sensitivity analyses. Normality of distributions was assessed using the Shapiro–Wilk test, which is more appropriate for smaller sample sizes than the Kolmogorov–Smirnov test. Continuous data with normal distributions were reported as means (±standard deviation) and analyzed using Student’s *t*-test. Continuous data with non-normal distributions were reported as median values and interquartile ranges (IQR) and analyzed using the Mann–Whitney U test. All between-group differences received 95% CI confidence interval calculations. Categorical variables were compared using Pearson’s Chi-square test or Fisher’s exact test when expected frequencies were small. The analysis included stratified tests for diagnosis to evaluate interaction effects while using likelihood ratio tests to determine interaction *p*-values.

The researchers conducted multivariable logistic and linear regression analyses to control for confounding variables which included lesion depth from pleura and lesion size and presence of pleural adhesions and emphysema severity and diagnosis and drainage device type. The researchers used linear regression to analyze total hospitalization costs because cost operated as a continuous dependent variable. The researchers used logistic regression models to analyze binary clinical results which included air leak duration exceeding two days and drainage time exceeding three days and complications and prolonged air leak and hospital stay exceeding four days. The analysis models used surgical technique as the main exposure factor while including all listed covariates. The researchers used variance inflation factors to check for multicollinearity (VIF < 2.5) and Hosmer–Lemeshow goodness-of-fit testing (*p* > 0.05) to evaluate model diagnostics for logistic models. 

The researchers performed sensitivity tests to determine how selection bias affected their research results. The Rosenbaum bounds analysis evaluated the necessary amount of hidden bias to change study results by using Γ values between 1.0 (no hidden bias) and 2.0 (substantial hidden bias). The researchers performed simulation tests which adjusted the pleural depth difference between groups by ±3 mm to determine its effect on effect size calculations. The research methods enable scientists to determine the extent that unmeasured confounding factors affect the observed research relationships.

The researchers established *p* < 0.05 as their statistical significance threshold. The researchers performed no multiple comparison adjustments for their two primary endpoints but treated all secondary endpoint results as exploratory findings. The researchers present complete *p*-values in their results to enable readers to determine their own significance thresholds.

### 2.10. Data Availability and Transparency

The anonymized dataset together with analysis code for producing study results can become available through the corresponding author after a reasonable request but only after following institutional data sharing rules and patient privacy laws. The research findings remain transparent and reproducible because readers can execute the provided code and de-identified dataset to reproduce all analyses.

## 3. Results

### 3.1. Patient Characteristics and Study Population

Between 2018 and 2024, a total of 134 patients underwent VATS wedge resection at our institution. Based on the predefined exclusion criteria, 59 patients were included in the study. Excluded patients consisted of those diagnosed with primary lung cancer requiring further anatomical lung resection (n = 40), patients with significant concurrent illnesses or severe pulmonary conditions (n = 18), those with a history of oncologic treatment (n = 10), and cases requiring conversion to thoracotomy (n = 7).

The study population was divided into two groups: the stapler group (S-group, n = 27) and the clamp-and-suture group (C-group, n = 32). Baseline demographic and clinical characteristics were comparable between the two groups. There were no statistically significant differences in patient age (*p* = 0.122), sex distribution (*p* = 0.636), smoking history (*p* = 0.483), presence of COPD (*p* = 0.893), pulmonary function (FEV1, *p* = 0.777), or indication for wedge resection (*p* = 0.103). The number of wedge resections performed per patient was also similar between the groups (*p* = 0.652) (Table 1 and Table 2).

### 3.2. Lesion Characteristics

Analysis of lesion characteristics revealed that the clamp-and-suture group had lesions significantly closer to the pleural surface (median 5 mm, IQR: 4) compared to the stapler group (median 8 mm, IQR: 6, *p* = 0.048). Lesion size was comparable between groups (stapler: median 18 mm, IQR: 12; clamp–suture: median 15 mm, IQR: 10; *p* = 0.234). Pleural adhesions were present in seven patients (25.9%) in the stapler group and five patients (15.6%) in the clamp–suture group (*p* = 0.332). The severity of emphysema was similar between groups, with moderate emphysema present in five patients (18.6%) in the stapler group and four patients (12.5%) in the clamp–suture group (*p* = 0.641). The stapler group used a median of two cartridges per case (range: 1–3) (Table 2).

### 3.3. Operative Findings

**Figure 3:**
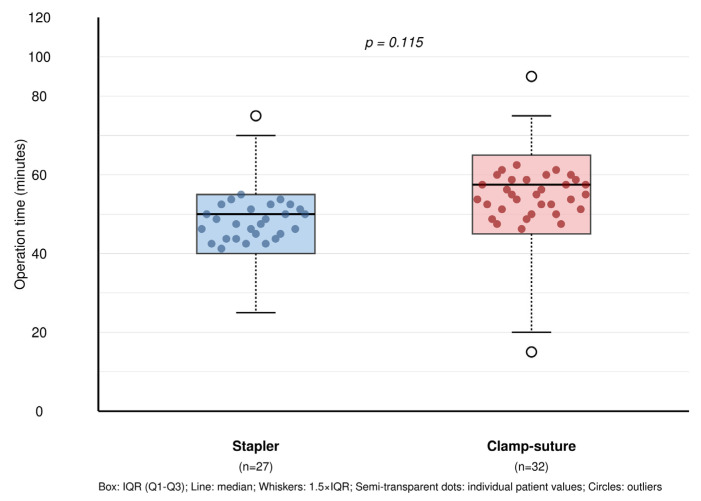
Operative time comparison between surgical techniques with overlaid individual patient data. Box plots show the distribution of operative times for stapler (n = 27) versus clamp–suture (n = 32) groups in VATS wedge resection. Semi-transparent dots represent individual patient values, demonstrating within-group variability. No significant difference was observed between groups (*p* = 0.115). Box: IQR (Q1–Q3); line: median; whiskers: 1.5 × IQR; semi-transparent dots: individual patient values; circles: outliers.

The median operation time was 10 min longer in the C-group (70 min; IQR: 20, 95% CI: 65–75) compared to the S-group (60 min; IQR: 15, 95% CI: 55–65), though this difference did not reach statistical significance (*p* = 0.115) (Figure 3). This increase in operative time in the C-group was attributed to the additional time required for meticulous suturing of the lung parenchyma following wedge resection. Drainage system selection showed no significant difference between groups (*p* = 0.824), with 24 Fr chest tubes used in 18 patients (66.7%) in the S-group and 20 patients (62.5%) in the C-group, while 12 Fr Hemovac drains were used in 9 patients (33.3%) and 12 patients (37.5%), respectively. The operating room time cost difference of $7 per hour between groups was statistically tested (*p* = 0.123) and did not reach significance.

### 3.4. Postoperative Outcomes

There was no statistically significant difference between the two groups in terms of air leak duration (median 2 days in both groups, difference 0 days, 95% CI: −0.5 to 0.5, *p* = 0.872). The median postoperative drainage time was 3 days in the S-group and 2 days in the C-group, with a difference of 0.5 days (95% CI: −0.2 to 1.2), but this difference was not statistically significant (*p* = 0.176). Overall, 12 patients (20.3%) developed postoperative complications, with no significant difference between the two groups (*p* = 0.741).

Complications were classified according to the Clavien–Dindo system: Grade I complications occurred in three patients (11.1%) in the S-group and four patients (12.5%) in the C-group; Grade II in two patients (7.4%) vs. one patient (3.1%); and Grade IIIa in one patient (3.7%) vs. one patient (3.1%), respectively (*p* = 0.823). Prolonged air leak (defined as air leak persisting beyond postoperative day 7) occurred in three patients (11.1%) in the S-group and four patients (12.5%) in the C-group, showing no significant difference (*p* = 1.000). Residual pleural space was observed in two patients (7.4%) in the S-group and two patients (6.3%) in the C-group (*p* = 1.000). Reinsertion of a chest tube was required in two patients in each group due to unresolved pleural effusion or persistent air leaks (*p* = 1.000). The median length of hospital stay was 4 days in both groups (difference 0 days, 95% CI: −0.8 to 0.8, *p* = 0.981). No patient required readmission within 30 days following discharge (Table 3).

### 3.5. Stratified Analysis by Diagnosis

The researchers conducted stratified analysis based on diagnosis to assess how surgical methods interact with different medical conditions. The analysis of all outcome measures revealed no significant interaction effects between surgical technique and diagnosis (*p* for interaction: 0.672 for operation time, 0.456 for air leak duration, 0.523 for drainage time, and 0.945 for complications). The stapler group required 55 min (IQR: 12) to complete operations for pneumothorax patients (n = 25) while the clamp–suture group needed 65 min (IQR: 15) (*p* = 0.089). The two surgical techniques produced equivalent results for patients with solitary pulmonary nodules (n = 13) and lung metastases (n = 21). The cost difference between stapler and clamp–suture techniques proved significant in every diagnostic subgroup (*p* < 0.001 for pneumothorax, *p* = 0.002 for solitary nodules, *p* < 0.001 for metastases) (Table 4).

### 3.6. Cost Analysis

A major finding of this study was the significant cost difference between the two surgical techniques. The study reports all costs in 2024 United States dollars (USD) while maintaining constant unit costs from the beginning to the end of the research period. The median total hospitalization cost in the S-group was $371.7 (IQR: 85), whereas it was significantly lower at $191.6 (IQR: 75) in the C-group (difference $180.1, 95% CI: 165–195, *p* < 0.001). The detailed unit cost breakdown appears in Table 3 which shows stapler cartridges at $180 per unit and suture materials at $12 per case and operating room time at $45 per hour and ward stay at $85 per day and chest tube management at $12 per day and laboratory tests at $15 per set and chest X-rays at $12 per exam.

The stapler cartridges in the stapler group cost $180 (IQR: 60) per case on average which made up 48.4% of the total hospital expenses. The cost analysis of stapler cartridges showed a straight correlation with the number of cartridges needed because patients who needed one cartridge paid $180 while those who needed two cartridges paid $360 and those who needed three cartridges paid $540. The surgical material expenses increased by $180 for each additional cartridge used. The suture materials used in the clamp–suture group had a total cost of $12 (IQR: 5). The operating room time expenses between groups showed a minimal difference of $7 per hour although the clamp–suture group spent more time in the operating room. The significant reduction in surgical material expenses compensated for the slightly longer operating room time in the clamp–suture group. The costs for ward stay and chest tube management and laboratory tests and chest X-rays remained equivalent between the two groups (Table 3). This finding highlights the economic advantage of using the clamp-and-suture technique over staplers, particularly in settings where disposable surgical instruments contribute substantially to healthcare costs.

### 3.7. Multivariable Regression Analysis

The researchers conducted multivariable regression analyses for all primary and secondary outcomes to address confounding variables and selection bias (Table 5). The cost-effectiveness of the clamp–suture technique proved highly significant after adjusting for lesion depth from pleura and additional factors including lesion size and presence of pleural adhesions and emphysema severity and diagnosis and drainage device type (−$172.4, 95% CI: −189 to −156, *p* < 0.001). The analysis showed that deeper lesions near the pleura resulted in cost reductions of $8.2 per millimeter (*p* < 0.001) but lesion size and adhesions and moderate emphysema levels led to higher costs ($3.1 per mm, *p* = 0.009 and $45.3, *p* = 0.001 and $52.1, *p* = 0.001, respectively).

The odds ratio for air leak duration exceeding two days between techniques showed no substantial difference after adjusting for other factors (OR 0.68, 95% CI: 0.13 to 3.51, *p* = 0.645). The analysis revealed that air leak duration extended with each millimeter of pleural distance (OR 1.18 per mm, *p* = 0.017) and with increasing lesion size (OR 1.09 per mm, *p* = 0.028) and when adhesions were present (OR 3.42, *p* = 0.031) and when emphysema was moderate (OR 4.21, *p* = 0.018). The adjusted analysis showed no meaningful differences between surgical approaches for drainage time and other complications.

### 3.8. Sensitivity Analysis for Selection Bias

The study’s non-randomized design and substantial pleural distance difference between groups required a sensitivity analysis to evaluate selection bias effects (Table 6). The cost benefit analysis using Rosenbaum bounds analysis demonstrated continued significance when researchers introduced substantial hidden bias (Γ = 2.0, cost difference -$148.2, *p* < 0.001). The simulation results demonstrated that the cost difference would remain significant at −142.7 dollars even when the groups had equal pleural depths. All sensitivity analyses produced non-significant results for clinical outcomes which indicate that the findings about air leak and drainage times are resistant to selection bias selection effects.

## 4. Discussion

The findings of this study suggest that the clamp-and-suture technique for wedge resection in video-assisted thoracoscopic surgery (VATS) may be a viable alternative to stapler use for carefully selected peripheral lesions. The results demonstrate comparable short-term outcomes in terms of air leak duration, postoperative drainage time, and complication rates while offering significant cost savings. The results need interpretation as initial findings which generate hypotheses instead of serving as final practice guidelines because our study design includes major methodological weaknesses from its retrospective non-randomized single-center single-surgeon approach. These findings are particularly relevant in healthcare settings where the financial burden of disposable surgical instruments is a concern.

### 4.1. Economic Analysis

One of the key advantages of staplers is their efficiency in providing a quick and secure closure, reducing intraoperative time and minimizing intraoperative air leaks [7,8]. However, this benefit comes at a substantial financial cost [6,7]. In contrast, the clamp-and-suture technique requires more time for parenchymal closure but offers significant economic benefits. The clamp-and-suture technique required a slightly longer operative time than stapler use during minimally invasive thoracoscopic procedures according to our study results (70 min vs. 60 min with *p* = 0.115). The current VATS literature supports this finding because stapler-free precision excision methods need longer surgical time for detailed dissection and precise suturing. The study by Suzuki et al. shows that RFID-guided precision excision for small lung nodules needs continuous real-time monitoring and manual suturing which lengthens surgical time but produces equivalent treatment results. The study by He et al. showed that lung-pro guided localization required longer wedge resection times than hook-wire techniques (16.4 min vs. 20.4 min) yet both methods delivered equivalent success rates and postoperative results. The clamp-and-suture technique required more surgical time but our research showed no differences in postoperative complications or hospital stay duration or drainage time between treatment groups [12,13]. This suggests that the added surgical time may be an acceptable trade-off given the substantial cost reduction for appropriately selected cases.

The economic implications of stapler use in thoracic surgery are substantial. In our cohort, the total hospitalization cost in the stapler group was nearly double that of the clamp-and-suture group ($371.7 vs. $191.6, *p* < 0.001), despite comparable clinical outcomes. The analysis adjustment maintained the cost benefit of the clamp–suture technique when accounting for confounding variables since the adjusted difference reached $172.4 (95% CI: −189 to −156, *p* < 0.001) (Table 5). The detailed cost analysis in Table 3 shows stapler cartridges cost $180 (IQR: 60) per case which made up 48.4% of total hospital expenses in stapler cases while patients needed two cartridges (range: 1–3) per case. The clamp-and-suture group required suture materials that cost $12 (IQR: 5). The cartridge price sensitivity analysis showed that the clamp–suture technique would remain cost-effective even when stapler prices dropped by 20% to $144 per cartridge because it would save $132 per case. The cost difference would increase to $228 per case when stapler prices rise by 20% to $216 per cartridge. The sensitivity analysis demonstrated that stapler expenses rise directly with cartridge consumption because each new cartridge adds $180 to the total expense which demonstrates the significant financial effect of stapler use during multiple wedge resections. This cost discrepancy is particularly relevant in resource-constrained healthcare systems where optimization of surgical expenditures is critical. The total hospitalization expenses per patient showed that stapler costs made up between 40% and 60% of the total expenses based on cartridge usage. The study results demonstrate how disposable surgical equipment creates substantial financial strain on patients.

The 2024 US dollar values in our cost analysis show direct surgical expenses and hospitalization costs which include materials and operating room time and drainage management and ward stay and laboratory tests and imaging as shown in Table 3. The prices of staplers differ between countries because different regions have distinct procurement rules and payment systems and market competition patterns. Stapler costs vary significantly across European markets, which may impact the calculations of relative cost-effectiveness and limit generalizability of our findings. As such, the cost-effectiveness of alternative techniques—such as clamp-and-suture—may differ across healthcare settings. Nonetheless, our preliminary results suggest that selective reduction in stapler use may potentially generate cost savings without compromising surgical safety or patient outcomes in carefully selected cases, thereby possibly enabling reallocation of resources to other critical areas of care.

### 4.2. Patient Selection and Technical Considerations

While the clamp-and-suture method presents clear potential advantages, it may not be suitable for all patients. The results of our regression analysis showed that deeper lesions and larger size and adhesions and moderate emphysema independently predicted both higher costs and longer air leak duration (Table 5) which confirms the need for proper patient selection. Deep-seated nodules, patients with severe emphysema, or cases involving extensive pleural adhesions may require the precision and reliability of staplers. The cost benefit of the clamp-and-suture technique appeared in all diagnostic groups according to Table 4 while operative times remained longer with this method for all conditions. Additionally, in cases where multiple wedge resections need to be performed within the same lobe, the stapler technique may offer more reproducible results. Therefore, patient selection remains a crucial factor in determining the appropriate surgical approach. In our limited experience, this method appeared most suitable for treating peripheral lesions which are located within 10 mm of the pleural surface and measure less than 20 mm in diameter when there are no substantial adhesions or emphysematous changes present.

### 4.3. Histopathological Considerations

Histopathological assessment is another important consideration when comparing these two techniques. Staplers create multiple rows of staples along the resection margin, which may complicate microscopic examination and histological interpretation [14,15,16,17]. The clamp-and-suture method provides better pathological assessment margins because it lacks stapler-related artifacts but our study did not gather data about margin quality or readability problems or re-excision rates. The theoretical benefit of this method needs confirmation through future studies which should include complete pathological assessment results. Future research needs to assess margin assessment quality through prospective evaluation of indeterminate margin rates and re-excision requirements and pathologist satisfaction scores between surgical methods. The assessment of margins becomes essential in suspected malignancies because proper margin evaluation determines treatment success.

### 4.4. Learning Curve and Technical Reproducibility

The clamp-and-suture method needs particular surgical expertise which surgeons might need time to learn after stapler-based surgical experience. The main technical obstacles in this procedure involve keeping proper suture tension and maintaining uniform spacing between sutures and preventing excessive tightening that could compromise aerostasis. The surgeon who performed the study had experience with both methods yet the time needed for surgeons to learn the clamp-and-suture technique after stapler-based surgery remains unknown. Based on our single-surgeon experience, the development of proficiency with this technique may require 10 to 15 supervised procedures, although this estimate needs confirmation through additional validation studies with objective assessment criteria. Training programs that want to implement this technique should develop formal educational programs with mentorship support. The study’s single-surgeon design creates a major challenge for external validity because individual surgeon skills and preferences strongly affect treatment results. The results need verification through multi-surgeon and multicenter studies to establish their reproducibility across various surgical teams and healthcare facilities.

### 4.5. Study Limitations, Strengths, and Future Directions

Our study has some important limitations. First and foremost, surgeons decided which technique to use during surgery—there was no pre-determined randomization. Naturally, we preferred the clamp–suture technique for lesions closer to the pleural surface (8 mm versus 5 mm on average). This makes it difficult to directly compare the two groups and may perhaps make the clamp–suture method appear safer than it is. Our multivariable regression analysis (Table 5) attempted to control for this selection bias by adjusting for lesion characteristics and other confounders. The sensitivity analysis (Table 6) showed that the cost benefit remains strong under significant hidden bias assumptions but clinical outcomes need careful evaluation because of the established selection bias.

Our sample of 59 cases was sufficient to show cost differences but insufficient to detect differences in complication rates. According to our calculations, we only had 40% power to detect a 15% difference in complications between the groups. All surgeries were performed by an experienced surgeon at a single center. The study results become less applicable to other settings because the research used one surgeon who worked at a single medical facility. The results reflect the experience of one surgeon at one institution and cannot be extrapolated to predict outcomes at other centers or with other surgeons. The learning curve for the clamp–suture method is also unclear—inexperienced surgeons may achieve different results.

We only followed up with patients for 30 days. We were unable to observe longer-term outcomes, which is a significant limitation. Nevertheless, our study has its strengths. Our 30-day follow-up was complete, we analyzed costs in detail, performed subgroup analyses based on diagnoses, and an experienced surgeon performed the operations using a standard technique. We provided preliminary information on how the clamp–suture method may work in specific patient groups and its potential cost-effectiveness within our institutional context. The study’s strengths fail to eliminate the basic flaws of its retrospective non-randomized design.

Future studies need to address these shortcomings. Randomized controlled trials, multicenter studies, training protocols, long-term follow-ups, pathological evaluations, and cost-effectiveness analyses in different healthcare systems should be conducted. Only then can we truly understand the value of this method.

## 5. Conclusions

Within the limitations of this single-center retrospective study, the clamp-and-suture method shows promise as a budget-friendly stapler replacement for peripheral lesions in VATS wedge resection when used in specific cases within resource-constrained environments. These results serve as initial exploratory findings which do not establish new clinical practices. The study results need interpretation with extreme caution because the research design involved retrospective data collection from non-randomized participants. The clamp-and-suture method reduces hospitalization expenses by $180.1 while delivering equivalent short-term results to stapling procedures for peripheral lesions situated near the pleural surface. The study’s results cannot and should not support immediate practice changes because it has several methodological weaknesses including selection bias and a small participant number (n = 59) and single-surgeon involvement and insufficient long-term monitoring. The non-randomized allocation process through intraoperative assessment created systematic differences between groups because simpler cases received the clamp-and-suture technique. The clamp-and-suture method demonstrates potential for treating peripheral lesions that are close to the pleura and measure less than 20 mm in diameter but needs verification through future clinical trials with specific patient selection rules and sufficient participant numbers and multiple surgeons at different centers and prolonged observation periods and detailed pathological evaluations to establish any clinical guidelines. The selection between stapler and clamp-and-suture techniques should currently be made on a case-by-case basis by considering lesion specifics and surgeon qualifications and hospital equipment availability. The stapler technique should continue as the standard treatment for most VATS wedge resections until future research provides strong evidence because the clamp–suture technique should only be used as an experimental procedure for specific cases by skilled surgeons.

## Figures and Tables

**Table 1:** Patients’ characteristics.

Variables	Data (n = 59)
**Age, year, median (IQR)**	53 (34)
**Sex, n (%)**	
** Women**	26 (44.1)
** Men**	33 (55.9)
**Cigarette use, n (%)**	41 (69.5)
Cigarette, pack/years, median (IQR) *	20 (20)
**Presence of COPD, n (%)**	18 (30.5)
**Diagnosis, n (%)**	
** Pneumothorax**	25 (42.4)
** Solitary pulmonary nodule**	21 (35.6)
** Lung metastasis**	13 (22.0)
**FEV1, liter, mean ± SD**	2.01 ± 1.01
**FEV1, %, mean ± SD**	81.6 ± 20.3
**Wedge resection type, n (%)**	
**Stapler**	27 (45.8)
**Clamp and suture**	32 (54.2)
**Number of wedge resection, n (%)**	
** 1**	54 (91.5)
** 2**	5 (8.5)
**Operation time, minute, median (IQR)**	70 (20)
**Postoperative suction applied, n (%)**	9 (15.3)
**Complication, n (%)**	12 (20.3)
**PAL prevalence, n (%)**	7 (11.9)
**Space prevalence, n (%)**	5 (8.5)
**Air leak duration, day, median (IQR)**	2 (1)
**Drainage time, day, median (IQR)**	2 (1)
**Length of hospital stay, day, median (IQR)**	4 (2)
**Total cost of hospitalization, dollar, median (IQR)**	208 (180)

* Calculated for the 41 patients who used cigarettes. Abbreviations: IQR, interquartile range; SD, standard deviation; COPD, chronic obstructive pulmonary disease; FEV1, forced expiratory volume in one second; PAL, prolonged air leak.

**Table 2:** Comparison of patient and lesion characteristics between groups.

Variables	Stapler (n = 27)	Clamp–Suture (n = 32)	*p* Value
**Patient Characteristics**			
**Age, year, median (IQR)**	55 (34)	51.5 (37)	0.122
**Sex, n (%)**			0.636
** Women**	11 (40.7)	15 (46.9)	
** Men**	16 (59.3)	17 (53.1)	
**Smoking (yes), n (%)**	20 (74.1)	21 (65.6)	0.483
Smoking amount, pack/years, median (IQR) *	20 (30)	17 (23)	0.358
**Presence of COPD, n (%)**	8 (29.6)	10 (31.3)	0.893
**Diagnosis, n (%)**			0.103
** Pneumothorax**	15 (55.6)	10 (31.3)	
** Solitary pulmonary nodule**	6 (22.2)	7 (21.9)	
** Lung metastasis**	6 (22.2)	15 (46.9)	
**FEV1, liter, mean ± SD**	2.08 ± 0.72	2.13 ± 1.06	0.404
**FEV1, %, mean ± SD**	80.5 ± 21.6	82.4 ± 19.7	0.777
**Number of wedge resections, n (%)**			0.652
** 1**	24 (88.9)	30 (93.8)	
** 2**	3 (11.1)	2 (6.2)	
**Lesion Characteristics**			
**Localization of wedge resection, n (%)**			0.897
** Upper lobe**	15 (55.6)	18 (56.2)	
** Middle lobe**	3 (11.1)	3 (9.3)	
** Lower lobe**	9 (33.3)	11 (34.3)	
**Lesion size, mm, median (IQR)**	18 (12)	15 (10)	0.234
**Distance from pleura, mm, median (IQR)**	8 (6)	5 (4)	0.048
**Presence of pleural adhesions, n (%)**	7 (25.9)	5 (15.6)	0.332
**Emphysema severity score, n (%)**			0.641
** None**	12 (44.4)	16 (50.0)	
** Mild**	10 (37.0)	12 (37.5)	
** Moderate**	5 (18.6)	4 (12.5)	
**Operative Details**			
**Operation time, minute, median (IQR) [95% CI]**	60 (15) [55–65]	70 (20) [65–75]	0.115
**Number of stapler cartridges used, median (range)**	2 (1–3)	-	-
**Drainage system used, n (%)**			0.824
** 24 Fr chest tube**	18 (66.7)	20 (62.5)	
** 12 Fr Hemovac drain**	9 (33.3)	12 (37.5)	
**Suture material cost, USD, median (IQR)**	-	12 (5)	-

* Calculated for the 41 patients who used cigarettes. Abbreviations: IQR, interquartile range; SD, standard deviation; CI, confidence interval; COPD, chronic obstructive pulmonary disease; FEV1, forced expiratory volume in one second; USD, United States dollars.

**Table 3:** Postoperative outcomes and cost analysis.

Variables	Stapler (n = 27)	Clamp–Suture (n = 32)	Difference [95% CI]	*p* Value
**Clinical Outcomes**				
**Postoperative suction applied, n (%) [95% CI]**	4 (14.8) [4.2–25.4]	5 (15.6) [5.3–26.0]	-	1.000
**Overall complications, n (%) [95% CI]**	6 (22.2) [9.6–34.8]	6 (18.8) [7.2–30.3]	-	0.741
**Complication severity (Clavien–Dindo), n (%)**				0.823
** Grade I**	3 (11.1)	4 (12.5)		
** Grade II**	2 (7.4)	1 (3.1)		
** Grade IIIa**	1 (3.7)	1 (3.1)		
**PAL prevalence, n (%) [95% CI]**	3 (11.1) [2.4–19.8]	4 (12.5) [3.5–21.5]	-	1.000
**Residual space, n (%) [95% CI]**	2 (7.4) [0–15.4]	2 (6.3) [0–13.2]	-	1.000
**Air leak duration, day, median (IQR)**	2 (1)	2 (1)	0 [−0.5 to 0.5]	0.872
**Drainage time, day, median (IQR)**	3 (2)	2 (2)	0.5 [−0.2 to 1.2]	0.176
**Chest tube reinsertion, n (%) [95% CI]**	2 (7.4) [0–15.4]	2 (6.3) [0–13.2]	-	1.000
**Length of hospital stay, day, median (IQR)**	4 (2)	4 (2)	0 [−0.8 to 0.8]	0.981
**30-day readmission, n (%)**	0 (0)	0 (0)	-	NA
**Cost Analysis (2024 USD)**				
**Unit Costs:**				
** Stapler cartridge (per unit)**	180	-		-
** Suture material (per case)**	-	12		-
** OR time (per hour)**	45	45		-
** Ward stay (per day)**	85	85		-
** Chest tube management (per day)**	12	12		-
** Laboratory tests (per set)**	15	15		-
** Chest X-ray (per exam)**	12	12		-
**Surgical materials, USD, median (IQR)**				
** Stapler cartridges (per case)**	180 (60)	-		-
** Suture materials**	-	12 (5)		-
**Cost by cartridge number, USD, median (IQR)**				
** 1 cartridge (n = 8, 29.6%)**	180 (0)	-		-
** 2 cartridges (n = 12, 44.4%)**	360 (0)	-		-
** 3 cartridges (n = 7, 25.9%)**	540 (0)	-		-
**Operating room time cost, USD, median (IQR)**	45 (10)	52 (12)	7 [2–12]	0.123
**Ward stay cost, USD, median (IQR)**	255 (60)	255 (60)	0 [−15 to 15]	1.000
**Chest tube management, USD**	35 (10)	30 (8)	5 [−2 to 12]	0.234
**Laboratory tests, USD**	15 (5)	15 (5)	0 [−2 to 2]	0.956
**Chest X-rays, USD**	12 (3)	12 (3)	0 [−1 to 1]	1.000
**Total hospitalization cost, USD, median (IQR)**	**371.7 (85)**	**191.6 (75)**	**180.1 [165–195]**	**<0.001**
**Stapler cost as % of total**	48.4%	-		-

Bold indicates statistical significance (*p* < 0.05). Abbreviations: CI, confidence interval; IQR, interquartile range; PAL, prolonged air leak; NA, not applicable; USD, United States dollars (2024 values). Unit costs remained fixed throughout the study period.

**Table 4:** Stratified analysis by diagnosis.

Outcomes	Pneumothorax			Solitary Nodule			Metastasis			*p* for Interaction
	Stapler (n = 15)	Clamp (n = 10)	*p*	Stapler (n = 6)	Clamp (n = 7)	*p*	Stapler (n = 6)	Clamp (n = 15)	*p*	
**Operation time, min, median (IQR)**	55 (12)	65 (15)	0.089	65 (18)	70 (20)	0.412	70 (20)	75 (22)	0.523	0.672
**Air leak duration, day, median (IQR)**	2 (1)	2.5 (1)	0.234	1 (1)	1 (0.5)	0.876	2 (1.5)	2 (1)	0.912	0.456
**Drainage time, day, median (IQR)**	3 (2)	3 (2)	0.812	2 (1)	2 (1)	0.934	3 (2)	2 (1.5)	0.234	0.523
**Complications, n (%)**	4 (26.7)	3 (30.0)	1.000	1 (16.7)	1 (14.3)	1.000	1 (16.7)	2 (13.3)	1.000	0.945
**Hospital stay, day, median (IQR)**	4 (2)	4.5 (2)	0.567	3 (1)	3 (1)	0.845	4 (2)	4 (2)	0.923	0.812
**Cost, USD, median (IQR)**	365 (80)	195 (70)	<0.001	375 (85)	185 (65)	0.002	380 (90)	190 (80)	<0.001	0.923

Abbreviations: IQR, interquartile range; USD, United States dollars (2024).

**Table 5:** Multivariable regression analysis for primary and secondary outcomes.

Outcomes	Unadjusted Analysis		Adjusted Analysis †	
	OR/β (95% CI)	*p* Value	OR/β (95% CI)	*p* Value
**Primary Outcomes**				
**Total hospitalization cost (USD)** **‡**				
**Clamp–suture vs** **. stapler**	−180.1 (−195 to −165)	<0.001	−172.4 (−189 to −156)	<0.001
**Distance from pleura (per mm)**	-	-	−8.2 (−12.1 to −4.3)	<0.001
**Lesion size (per mm)**	-	-	3.1 (0.8 to 5.4)	0.009
**Presence of adhesions**	-	-	45.3 (18.2 to 72.4)	0.001
**Moderate emphysema**	-	-	52.1 (21.3 to 82.9)	0.001
**Air leak duration > 2 days**				
**Clamp–suture vs** **. stapler**	1.14 (0.25 to 5.18)	0.872	0.68 (0.13 to 3.51)	0.645
**Distance from pleura (per mm)**	-	-	1.18 (1.03 to 1.35)	0.017
**Lesion size (per mm)**	-	-	1.09 (1.01 to 1.18)	0.028
**Presence of adhesions**	-	-	3.42 (1.12 to 10.45)	0.031
**Moderate emphysema**	-	-	4.21 (1.28 to 13.85)	0.018
**Secondary Outcomes**				
**Drainage time > 3 days**				
**Clamp–suture vs** **. stapler**	0.67 (0.23 to 1.94)	0.456	0.42 (0.12 to 1.47)	0.176
**Distance from pleura (per mm)**	-	-	1.15 (1.02 to 1.30)	0.023
**Lesion size (per mm)**	-	-	1.07 (0.99 to 1.15)	0.089
**Any complication**				
**Clamp–suture vs** **. stapler**	0.82 (0.24 to 2.77)	0.741	0.58 (0.14 to 2.41)	0.452
**Distance from pleura (per mm)**	-	-	1.12 (0.98 to 1.28)	0.097
**Presence of adhesions**	-	-	2.87 (0.89 to 9.25)	0.078
**Prolonged air leak (>7 days)**				
**Clamp–suture vs** **. stapler**	1.14 (0.23 to 5.73)	0.872	0.71 (0.12 to 4.21)	0.703
**Distance from pleura (per mm)**	-	-	1.21 (1.04 to 1.41)	0.014
**Moderate emphysema**	-	-	5.12 (1.34 to 19.58)	0.017
**Hospital stay > 4 days**				
**Clamp–suture vs** **. stapler**	0.98 (0.35 to 2.74)	0.970	0.62 (0.19 to 2.03)	0.429
**Any complication**	-	-	8.34 (2.31 to 30.12)	0.001
**Drainage device (24 Fr vs** **. 12 Fr)**	-	-	2.15 (0.71 to 6.51)	0.174

† Adjusted for lesion depth from pleura, lesion size, presence of pleural adhesions, emphysema severity, diagnosis, and drainage device type; ‡ Linear regression coefficient (β); all others are logistic regression odds ratios (OR). Abbreviations: OR, odds ratio; CI, confidence interval; USD, United States dollars (2024).

**Table 6:** Sensitivity analysis for selection bias impact.

Analysis	Cost Difference (USD)	Air Leak OR	Drainage OR
**Base case (observed)**	−180.1	0.68	0.42
**Rosenbaum bounds analysis**			
**Γ = 1.0 (no hidden bias)**	−180.1	0.68	0.42
**Γ = 1.5**	−165.3	0.82	0.54
**Γ = 2.0**	−148.2	1.03	0.71
**Pleural depth simulation**			
**Depth difference −3 mm** *	−156.4	0.95	0.63
**Depth difference 0 mm**	−142.7	1.18	0.81
**Depth difference +3 mm**	−128.9	1.45	1.04

* Negative values indicate clamp–suture lesions closer to pleura (as observed); Γ = odds of differential assignment to treatment due to unobserved factors interpretation: cost benefit remains significant (*p* < 0.001) even at Γ = 2.0; clinical outcomes show no significant difference remains robust to moderate selection bias.

## Data Availability

The data presented in this article cannot be shared publicly to ensure privacy of the individuals who participated in the study. However, the data can be shared upon reasonable request from the corresponding author.

## Abbreviations

COPD	Chronic obstructive pulmonary disease
CT	Computed tomography
IQR	Interquartile range
SPSS	Statistical Package for the Social Sciences
VATS	Video-assisted thoracoscopic surgery
